# Robust Sub-nanomolar Library Preparation for High Throughput Next Generation Sequencing

**DOI:** 10.1186/s12864-018-4677-y

**Published:** 2018-05-04

**Authors:** Wells W. Wu, Je-Nie Phue, Chun-Ting Lee, Changyi Lin, Lai Xu, Rong Wang, Yaqin Zhang, Rong-Fong Shen

**Affiliations:** 1 Facility for Biotechnology Resources, Center for Biologics Evaluation and Research, Food and Drug Administration, 10903 New Hampshire Avenue, Silver Spring, MD 20993 USA; 2 OBP/DBRR-III, Center for Drug Evaluation and Research, Food and Drug Administration, Silver Spring, MD 20993 USA

**Keywords:** Next generation sequencing, Illumina, MiSeq, HiSeq, Sub-nanomolar libraries

## Abstract

**Background:**

Current library preparation protocols for Illumina HiSeq and MiSeq DNA sequencers require ≥2 nM initial library for subsequent loading of denatured cDNA onto flow cells. Such amounts are not always attainable from samples having a relatively low DNA or RNA input; or those for which a limited number of PCR amplification cycles is preferred (less PCR bias and/or more even coverage). A well-tested sub-nanomolar library preparation protocol for Illumina sequencers has however not been reported. The aim of this study is to provide a much needed working protocol for sub-nanomolar libraries to achieve outcomes as informative as those obtained with the higher library input (≥ 2 nM) recommended by Illumina’s protocols.

**Results:**

Extensive studies were conducted to validate a robust sub-nanomolar (initial library of 100 pM) protocol using PhiX DNA (as a control), genomic DNA (*Bordetella bronchiseptica* and microbial mock community B for 16S rRNA gene sequencing), messenger RNA, microRNA, and other small noncoding RNA samples. The utility of our protocol was further explored for PhiX library concentrations as low as 25 pM, which generated only slightly fewer than 50% of the reads achieved under the standard Illumina protocol starting with > 2 nM.

**Conclusions:**

A sub-nanomolar library preparation protocol (100 pM) could generate next generation sequencing (NGS) results as robust as the standard Illumina protocol. Following the sub-nanomolar protocol, libraries with initial concentrations as low as 25 pM could also be sequenced to yield satisfactory and reproducible sequencing results.

**Electronic supplementary material:**

The online version of this article (10.1186/s12864-018-4677-y) contains supplementary material, which is available to authorized users.

## Background

Most high throughput DNA sequencing facilities use Illumina sequencers such as HiSeq or MiSeq. Current Illumina protocols for sample loading onto the flow cells call for ≥2 nM denatured cDNA libraries. In standard protocols, 2–4 nM libraries are first denatured by adding an equal volume of NaOH (0.2 N or 0.1 N, respectively, in MiSeq or HiSeq). The denatured libraries are then diluted with a hybridization buffer (HT1) to a range of 4–20 pM for final loading onto the flow cells. NaOH here plays dual roles, one good and essential and the other not so desirable. At 50–100 mM, NaOH effectively denatures double-strand cDNA to form single-strand DNA, but its presence also inhibits hybridization of the single-strand DNA library to oligonucleotides anchored on the flow cells, thus decreasing the density of clusters formed after bridge amplification. Therefore Illumina protocols consist of a step of diluting libraries, from nM to pM, to reduce the final concentration of NaOH (≤ 1 mM) in the libraries before loading onto the flow cells.

While the current protocols are adequate for samples with adequate amounts of starting material, there are occasions where the starting sample is severely limited (e.g., clinical biopsy specimens, single cell sequencing). In such situations, the existing protocols may not allow loading at the recommended concentrations. In other cases where PCR amplification may allow the use of the standard ≥2 nM protocol, a restricted number of PCR cycles would be preferred so as to minimize PCR-induced biases and/or uneven coverage. To meet these needs, the aim of our study was to devise and validate a protocol capable of robustly working with sub-nanomolar libraries to achieve results comparable to those obtained with Illumina’s current protocols. A survey of published work found that only scant literature offers tips and tricks for sub-nanomolar library loading. Quail M.A et al. [1] reported modified hybridization buffers in handling sub-nanomolar libraries, but did not present comparative results from different preparations. Lott, S.E. et al. [2] proposed modifying cBot cluster operations to sandwich the library between two air gaps and centered within the flow cell. This latter approach is neither straightforward nor universal, as it is not applicable to a MiSeq sequencer or the onboard loading using the HiSeq. In both cases, cluster amplification does not require the use of a cBot. In this report, we tested a simple protocol and demonstrated that very satisfactory and informative results are attainable with libraries 20X or even 80X less concentrated than the recommended 2 nM.

## Methods

### Samples

PhiX control standard (V3, 10 nM) was purchased from Illumina (San Diego, CA). Serially diluted loading samples of PhiX were sequenced on an Illumina HiSeq 2500 or MiSeq sequencer for 50 single-read cycles. Genomic DNAs from strain variants of *Bordetella bronchiseptica* (strain RB50, ATCC BAA-588) and *Bordetella pertussis* (strain Tohama I) were extracted using Qiagen (Germantown, MD) DNeasy Kit. Genomic DNA from microbial mock community B (20 bacterial strains with staggered rRNA operon counts from 1000 to 1000,000 copies/μl) for 16S rRNA gene sequencing was from Bei Resources (Manassas, VA). HCoEpiC cells were isolated from human colonic tissues (ScienCell Research Laboratories, Carlsbad, CA). HCT116 is a human colon cancer cell line (ATCC, Manassas, VA). Total RNAs of the HCoEpiC and HCT116 were extracted using the Qiagen RNeasy Mini Kit.

**Fig. 1:**
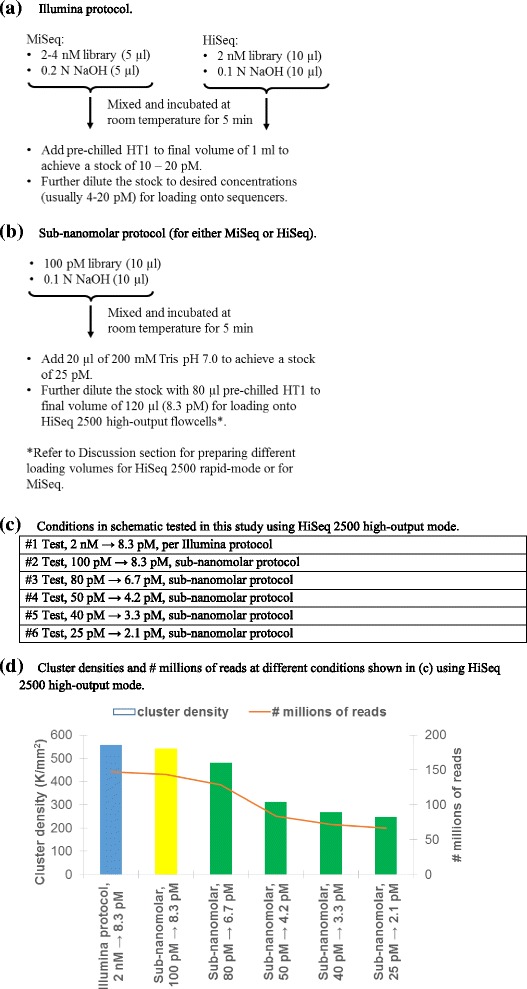
Details and comparisons of Illumina protocol and sub-nanomolar protocol. **a** Illumina protocol. **b** Sub-nanomolar protocol. **c** Conditions in schematic tested in this study. **d** Cluster densities and # millions of reads at different conditions shown in (**c**)

The standard Illumina protocol for nanomolar library preparation (2–4 nM) for HiSeq or MiSeq sequencers is presented in Fig. 1a. An alternative, a sub-nanomolar protocol we devised, is presented in Fig. 1b. The efficacy of our sub-nanomolar protocol, as compared to Illumina protocols, was validated in five comparative studies using different types of libraries. In the first study, sequencing results were compared between control PhiX DNA library, denatured following the Illumina protocol (as the baseline), and various concentrations of PhiX libraries prepared with the sub-nanomolar protocol. In the second study, genomic DNA libraries (*Bordetella bronchiseptica,* strain RB50 and *Bordetella pertussis* strain Tohama I), prepared according to the Illumina protocol (2 nM) and the sub-nanomolar protocol (100 pM) were denatured and diluted to 10 pM and sequenced on a MiSeq sequencer. In the third study, mRNA libraries, prepared according to the Illumina protocol (2 nM) and the sub-nanomolar protocol (100 pM), respectively, were denatured and diluted to 8.3 pM before loading onto the neighboring lanes in a flow cell and sequenced on a HiSeq 2500 sequencer. In the fourth study, small RNA libraries were prepared and compared under the same conditions as in the third study. In the fifth study, 16S rRNA gene sequencing was carried out under the aforementioned conditions (except for the final loading amount of 4 pM with 10% PhiX) for comparison using a MiSeq sequencer.

### Genomic DNA, mRNA, small RNA, and 16S metagenomic sample preparation and sequencing

The qualities of genomic DNA samples were assessed using a Nanodrop spectrophotometer (Thermo Scientific, Madison, WI) based on A260/280 and A260/A230. The qualities of RNA samples were assessed using Agilent (Santa Clara, CA) 2100 Bioanalyzer to ensure the RNA Integrity Number (RIN) of at least 8. Sample preparation protocols of DNA-sequencing, messenger RNA-sequencing, small RNA-sequencing, and 16S rRNA gene sequencing are briefly summarized below.

#### DNA-sequencing

Genomic DNA samples were processed following the protocol of Illumina TruSeq DNA PCR-Free Sample Preparation Kit. One microgram of gDNA sample was subjected to fragmentation, end repairing, size selection, adenylation tailing, and adapter ligation. Paired-end sequencing (150 × 2 cycles, V2 cartridge) of multiplexed cDNA samples was carried out on an Illumina MiSeq sequencer.

#### mRNA-sequencing

RNA samples were processed following the protocol for the Illumina TruSeq Stranded mRNA Sample Preparation Kit. Poly (A) tailed RNA was purified from 1 μg of total RNA, fragmented, and reverse-transcribed into cDNAs. Double strand cDNAs were adenylated at the 3′ ends and individually indexed, followed by limited-cycle (15) amplification. Paired-end sequencing (100 × 2 cycles) of multiplexed mRNA samples per lane was carried out on an Illumina HiSeq 2500 sequencer.

#### Small RNA-sequencing

RNA samples of HCoEpiC were processed following the protocol for the Illumina TruSeq Small RNA Sample Preparation Kit. In brief, RNA adapters specifically targeting the 3′ hydroxyl group or the 5′ monophosphate group were ligated to small RNA present in 1 μg total RNA. Adapter-modified small RNA was then subjected to reverse transcription to yield the first-strand cDNA. This was followed by synthesis of the second-strand cDNA and a limited-cycle (11) amplification, by which individual indexes were added. The small RNA libraries were loaded onto a Sage Science (Beverly, MA) Pippin Prep using 3% (to elute 145–160 bp for microRNA) or 2% agarose gel (to elute 160–320 bp for other small noncoding RNA). Single-end sequencing of multiplexed small RNA libraries was carried out on an Illumina HiSeq 2500 sequencer for 50- or 120-cycles, respectively, for microRNA and other small noncoding RNA.

#### 16S rRNA gene sequencing

Microbial mock community B samples were processed following the protocol for 16S Metagenomic Sequencing Library Preparation (Illumina, Part # 15044223 Rev. B). In brief, custom primer pairs specifically targeting variable V3 and V4 regions of the 16S rRNA gene were used to create a single amplicon of approximately ~ 460 bp. During the amplification using 2-staged PCR (25 and 8 cycles, respectively), Illumina sequencing adapters and dual-index barcodes were added to the amplicons. Paired-end sequencing (300 × 2 cycles, V3 cartridge) of multiplexed cDNA samples was carried out on an Illumina MiSeq sequencer.

### Data analyses

Fastq files obtained from MiSeq and HiSeq 2500 sequencers were generated using MiSeq Reporter v.2.6.2.3 and CASAVA v.1.8.2 (or later, bcl2fastq v.2.2.0), respectively. Only those reads passing the chastity filter were reported. Data analyses performed at our core facility are briefly summarized below, and data analyses of mRNA- and small RNA-sequencing were described previously [3].

#### DNA-sequencing

BWA v.0.7.9 was used to align reads in fastq files to the *Bordetella bronchiseptica* reference genome (NCBI accession: BX470250.1; gi|33,591,071) and *Bordetella pertussis* reference genome (NCBI accession: NC_002929.2; gi|33,591,275). GATK v.1.6.23 was used as a variant caller for SNPs and short indels. The above tasks were performed using wrappers in MiSeq Reporter. The program, deepTools v.2.5.0.1, was used to compare the genome-wide similarity of bam files by running the program in “bins” mode using default settings [4]. In short, “multiBamSummary” computes the read coverages for genomic regions for the paired (i.e. Illumina standard vs. sub-nanomolar protocols) bam files; followed by “plotCorrelation” to calculate and visualize pairwise correlation values between the read coverages.

#### mRNA-sequencing

Tophat v.2.1.0 was used to align reads in fastq files to the UCSC human hg38 reference genome. Cufflinks v.2.2.1 was used to assemble the transcriptome based on the hg38 reference annotation. Cuffdiff v.2.2.1 was used to calculate expression and test the statistical significance of observed differential expressions using default settings [5]. The quantification of relative abundance of each transcript was reported as Reads Per Kilobase per Million (RPKM) [6].

#### Small RNA-sequencing

First, Cutadapt 1.9.1 was used to trim Illumina TruSeq adapter sequences from fastq files at 3′ ends. Subsequently, for microRNA, miRDeep2 v.2.0.0.7 was used to map the reads to the miRBase (version 21) microRNA database and quantify microRNA expression levels. For other small noncoding RNA, data were analyzed using Qiagen (Germantown, MD) CLC Genomics Workbench 8.5.1 for mapping trimmed reads to the Ensemble GRCh38.82 noncoding RNA database and quantifying the levels of small RNA expression.

#### 16S rRNA gene sequencing

Illumina BaseSpace 16S Metagenomics app was used to generate a classification of reads at taxonomic levels from kingdom to species. The classification step uses a proprietary algorithm that provides species-level classifications for paired-end reads, involving matching short subsequences of the reads (called words) to a set of 16S reference sequences (Illumina-curated version of the Greengenes database). The accumulated word matches for each read were used to assign reads to a particular taxonomic classification.

## Results

The main issue using the Illumina’s buffer and protocol for a library < 2 nM is the high dilution factor required to reduce NaOH concentration from 50 to 100 mM (to denature dsDNA) down to ≤1 mM (suitable for loading). This is due mainly to the low-capacity buffer provided (HT1, proprietary composition), necessitating the use a large volume. Consequentially, the effective concentration of single-stranded DNA molecules in the library, for binding to oligo anchors on flow cells, is significantly reduced. In our sub-nanomolar protocol, a small volume of high-capacity buffer (20 μl of 200 mM Tris pH 7.0) is used after NaOH denaturing (Fig. 1b), allowing a near neutral pH condition for hybridization yet with minimal sample dilution (20 μl vs. 980–990 μl needed in Illumina protocol, Fig. 1a).

The efficacy of our sub-nanomolar protocol (Fig. 1b) as compared to the Illumina protocol (Fig. 1a) was validated in five comparative studies using: 1) Illumina PhiX control; 2) gDNA libraries; 3) mRNA libraries; 4) small RNA libraries; and 5) 16S V3 / V4 amplicons.

In the first comparative study, a range of Illumina PhiX concentrations, from 2 nM down to 25 pM, was used to assess the lowest library concentration that still yields reliable and reproducible sequencing output. Based on our experience, at least 100 pM cDNA could readily be obtained even for very challenging projects. Figure 1b thus was designed to test our sub-nanomolar protocol, based on a 100 pM library as the baseline. Following the steps in Fig. 1b, the final concentration for loading onto the flow cell was 8.3 pM, a concentration attainable for a variety of projects using either a HiSeq or MiSeq sequencer (typically 4–20 pM, as in Fig. 1a). The library as a baseline following the sub-nanomolar protocol is highlighted in yellow in Fig. 1c. A series of lower input PhiX DNA libraries (80–25 pM library before denaturing, corresponding to 6.7–2.1 pM loading) was further studied (green, Fig. 1c). The control library was prepared following the standard Illumina protocol (2 nM library, 8.3 pM loading; Fig. 1c, blue). Fig. 1d and Additional file 1: Table S1a show that the standard Illumina protocol (the control) and the sub-nanomolar protocol, both at 8.3 pM loading (the baseline), yielded similar cluster densities and # of reads. Fig. 1d and Additional file 1: Table S1a also demonstrate that satisfactory results were obtained with libraries which had been scaled down significantly from an initial concentration of 100 pM. For example, the one started with as low as 25 pM input library generated a read number only slightly less than half of that under the baseline condition (100 pM). Additional file 1: Table S1a further summarizes other run metrics: % of alignment to PhiX reference genome, % of error rate, and Q30 profiles, illustrating that the standard Illumina protocol and the sub-nanomolar protocol yielded highly comparable results.

**Fig. 2:**
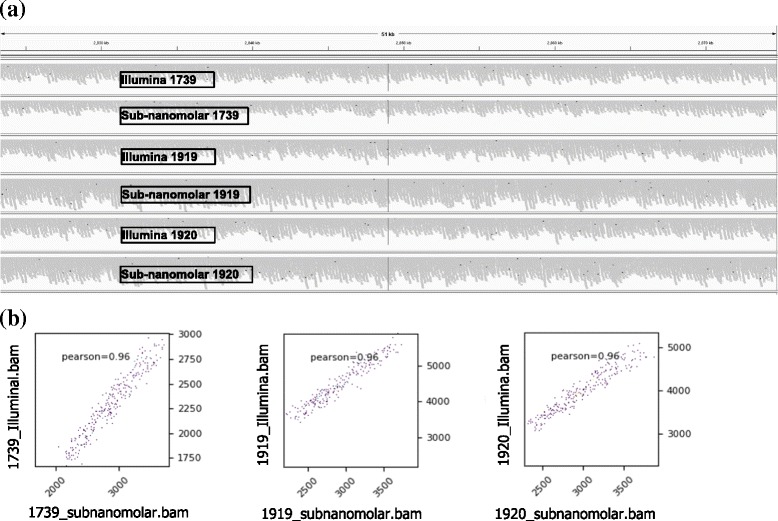
Similar patterns between Illumina protocol and sub-nanomolar protocol of the three *Bordetella bronchiseptica* strain RB50 samples: **a** read pile-ups in a snapshot of a random genomic region. **b** genome-wide similarities of read coverages using deepTool

In the second study of DNA-sequencing and mapping data of the three *Bordetella bronchiseptica* RB50 strains and one *Bordetella pertussis* Tohama I strain are summarized in Additional file 1: Table S1b and variant calling data in Additional file 2: Table S2. With initial library amounts of 20× less (the sub-nanomolar protocol, 100 pM → 10 pM) vs. the Illumina protocol (2 nM → 10 pM), the overall numbers of reads (7,969,530 vs. 7,116,714) and the average coverage depth (59 vs. 54) suggest that satisfactory (even slightly better, see Discussion) results are attainable with the sub-nanomolar protocol. In addition, Fig. 2a shows similar patterns of reads’ pile-ups in a snapshot of a random genomic region of the three *Bordetella bronchiseptica* RB50 strains between the two protocols. Fib. 2b demonstrates genome-wide similarities of read coverages using deepTools between the two protocols.

**Fig. 3:**
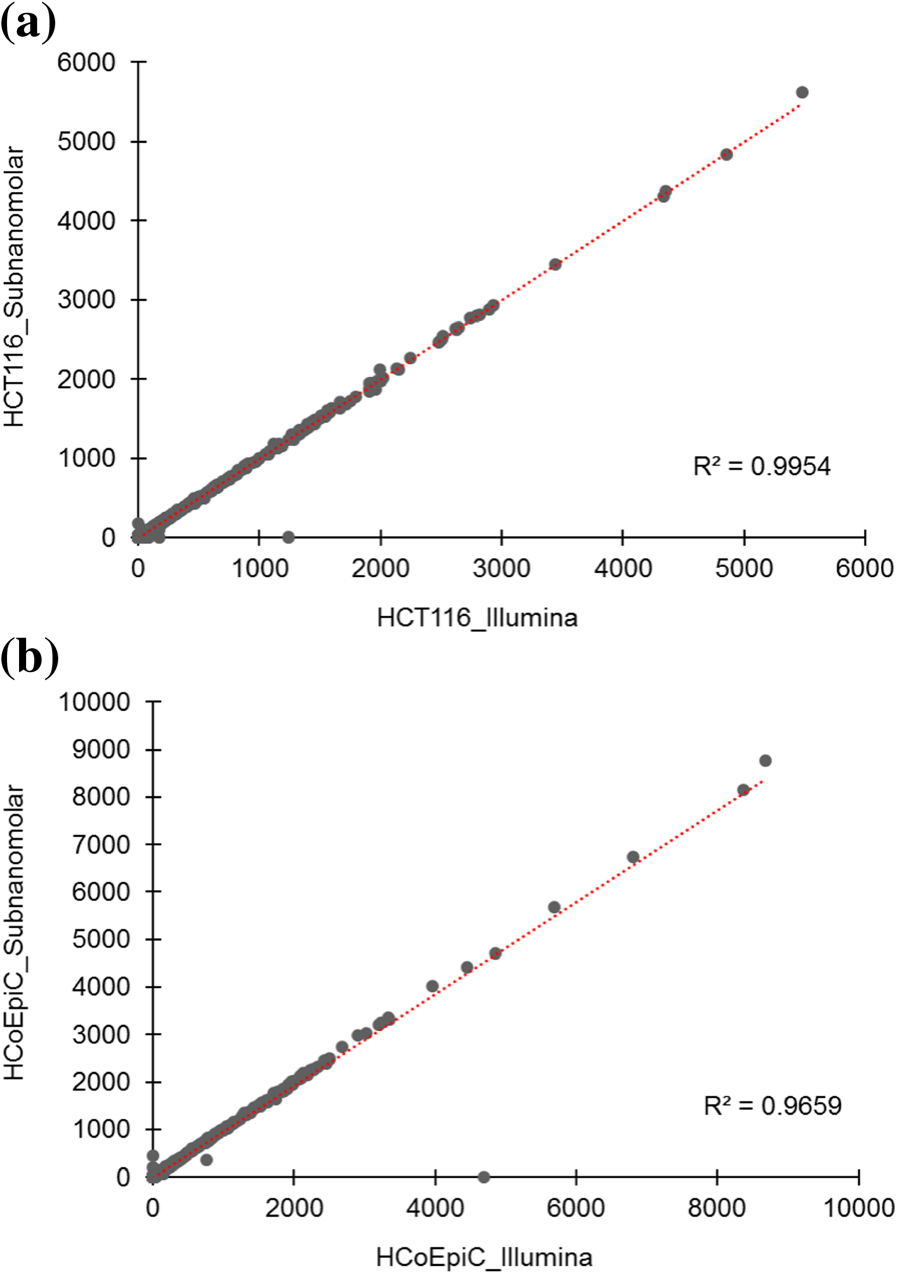
The comparative study (mRNA libraries) demonstrates the Illumina protocol (2 nM → 8.3 pM) and the sub-nanomolar protocol (100 pM → 8.3 pM) deliver highly comparable RPKM (x and y axis), using HCT116 (**a**) and HCoEpiC (**b**)

**Fig. 4:**
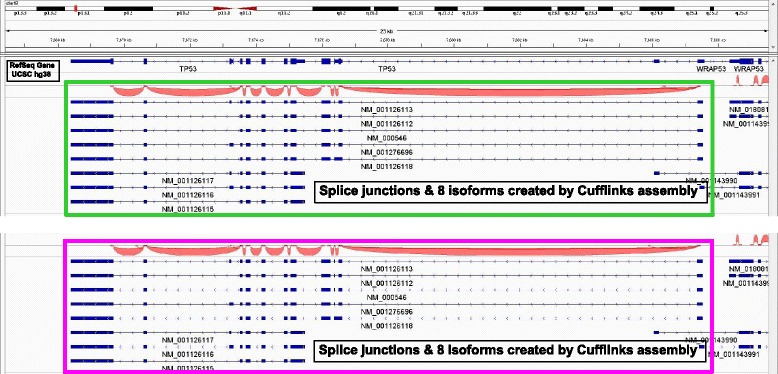
Visualization plots of TP53 transcript’s splice junctions (red) and eight isoforms (with gene exonic regions being blue solid blocks, connected by thin blue lines representing introns) are highly similar using Illumina protocol (top green rectangle) and sub-nanomolar protocol (bottom pink rectangle) using HCT116

The third study (mRNA libraries) demonstrated that the standard Illumina protocol (2 nM → 8.3 pM) and the sub-nanomolar protocol (100 pM → 8.3 pM) delivered highly comparable RPKM of a total of ~ 12,000 transcripts, as shown Fig. 3 (a) and (b) (data in Additional file 3: Table S3). Using TP53 gene of HCT116 as an example, visualization plots of splicing junctions and eight isoforms assembled by Cufflinks shown in Fig. 4 are also highly similar using Illumina protocol (top green rectangle) and sub-nanomolar protocol (bottom pink rectangle).

**Fig. 5:**
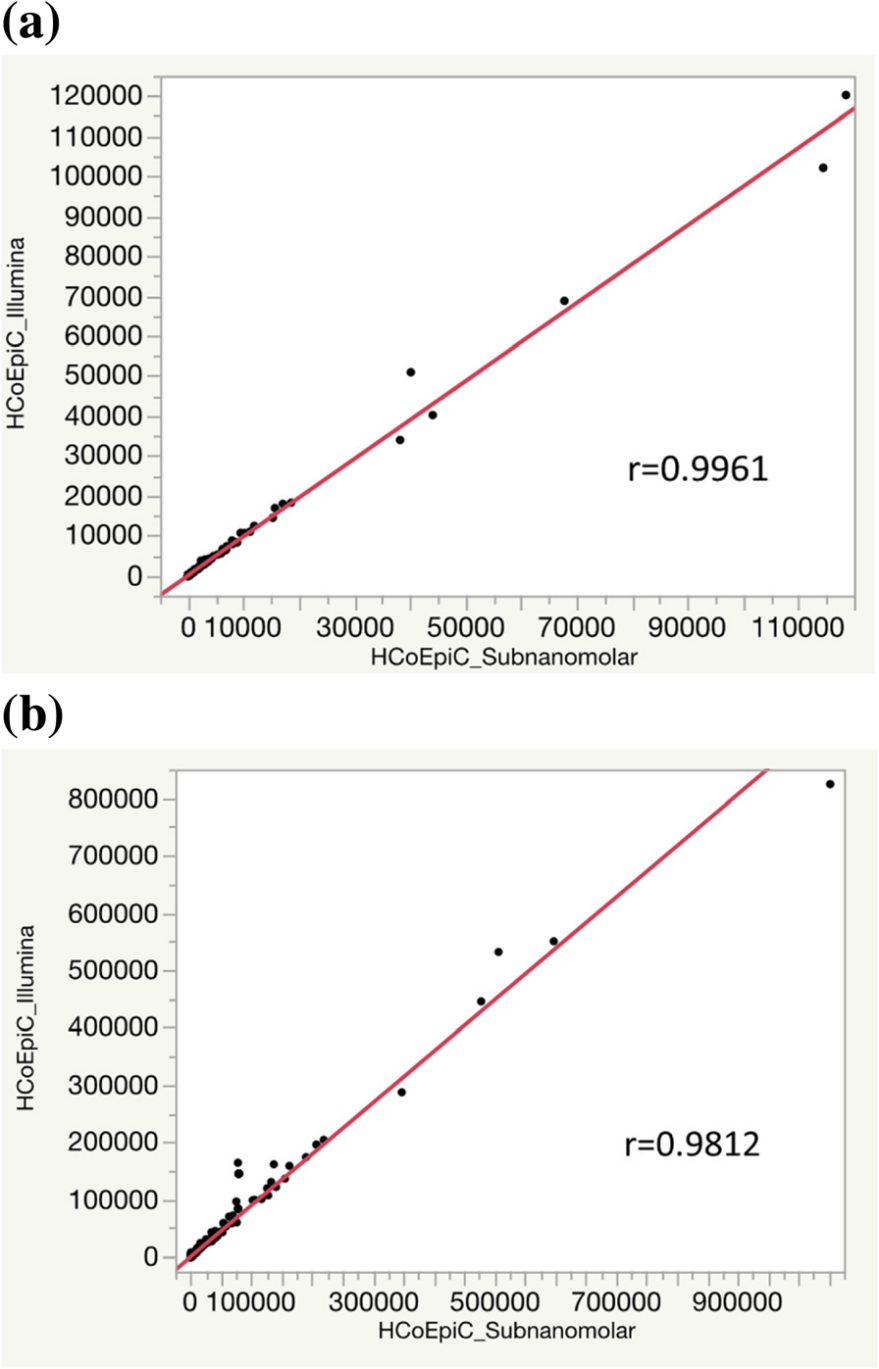
The comparative study (small RNA libraries of HCoEpiC) demonstrates the Illumina protocol (2 nM → 8.3 pM) and the sub-nanomolar protocol (100 pM → 8.3 pM) deliver highly comparable RPKM (x and y axis). **a** microRNA. **b** other small noncoding RNA

The fourth study (small RNA libraries) again demonstrated that comparable RPKM results were obtained with the sub-nanomolar loading protocol (100 pM → 8.3 pM) and the Illumina protocol (2 nM → 8.3 pM) as presented in Fig. 5. Data for microRNA and other small noncoding RNA can be found in Additional file 4: Tables S4 and Additional file 5: Table S5, respectively. NGS has revealed that microRNAs exist in multiple variants, abbreviated as isomiRs [7]. Following the TP53 example chosen above, p53 activates the transcription of miR-34 family (miR-34a, 34b, and 34c) [8]. The percentages of three isomiRs (5′ modifications, 3′ modifications, and nucleotide substitution) of the miR-34 family shown in Table 1 (summarized from Additional file 6: Table S6) further demonstrate that the sub-nanomolar loading yielded similar isomiR distributions comparable to that obtained from the Illumina protocol.

**Table 1:** Percentages of isomiRs (# of the isomiRs showing the indicated modification / # of total reads) of the miR-34 family activated by p53

	5′ modifications		3′ modifications		nucleotide substitution	
	Illumina	sub-nanomolar	Illumina	sub-nanomolar	Illumina	sub-nanomolar
hsa-miR-34a-5p	4.2%	3.2%	58.3%	58.0%	9.6%	9.8%
hsa-miR-34a-3p	~	~	~	~	~	~
hsa-miR-34b-5p	95.1%	96.3%	91.8%	92.6%	6.6%	7.4%
hsa-miR-34b-3p	73.4%	66.7%	98.7%	100.0%	7.6%	8.3%
hsa-miR-34c-5p	0.9%	1.4%	6.1%	5.2%	2.6%	2.3%
hsa-miR-34c-3p	~	~	~	~	~	~

~: # of -3p reads much lower than # of -5p reads to show reliable ratios

**Fig. 6:**
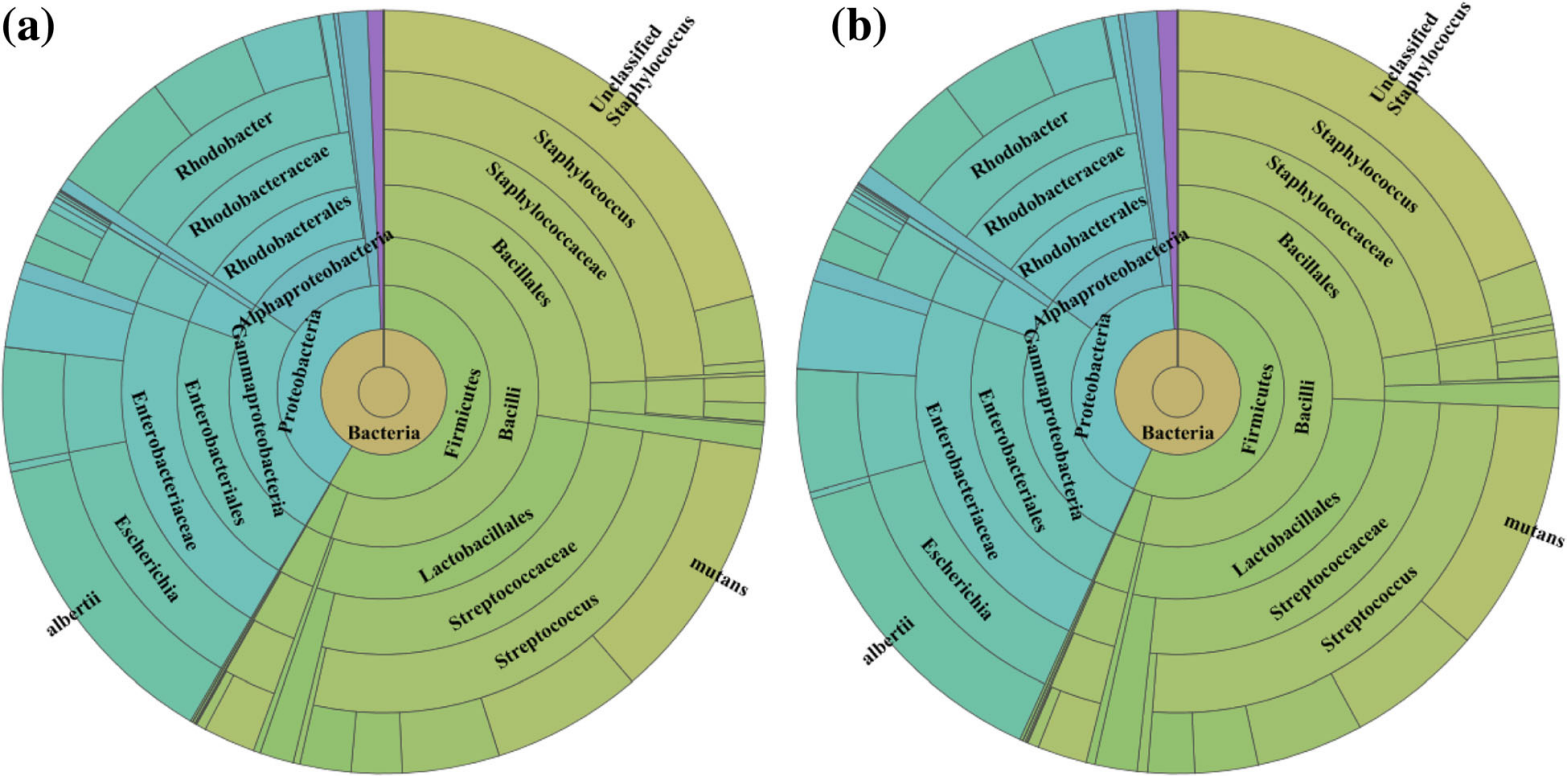
Sunburst charts show highly similar taxonomic hierarchy and relative abundance of classification of **a** the Illumina protocol (2 nM → 4 pM). **b** the sub-nanomolar protocol (100 pM → 4 pM)

In the fifth comparison using staggered concentrations of microbial mock community B, 16S rRNA gene sequencing showed that similar numbers of reads were generated from the Illumina protocol (2,249,926) and the sub-nanomolar protocol (2,148,506). Sunburst charts created using Illumina BaseSpace 16S Metagenomics app (Fig. 6) illustrate the highly similar taxonomic hierarchy and relative abundance of classification. Additional file 7: Table S7 (figure and data) further demonstrates the strong correlation (r^2^ of 0.9946) between the two workflows.

## Discussion

NGS libraries which do not meet the concentration threshold required by the Illumina protocol (≥ 2 nM) are often not submitted for sequencing analysis due to the lack of a success-proven working protocol. Given the cost of the reagent kits and other consumables and time required for analyzing the suboptimal fastq files, this is a reasonable concern. Our results in Fig. 1d show that even at 25 pM PhiX, the number of reads generated is only slightly less than half of that from the baseline condition (2 nM), suggesting that libraries of lower concentrations could still be sequenced and yield rather satisfactory outcomes. The lowest concentration of library (25 pM) reported in Fig. 1d was not the lowest level we tested. We tested down to 10 pM using HiSeq 2500 and still generated sequencing outputs proportional to those obtained from the standard (2 nM) loading. However, validation tests at this level (10 pM) using MiSeq on aliquots from the same preparation gave variable results. The reason is not clear at present. We suspect it might have to do with the stability of libraries at very low concentrations. The current Illumina protocol states that PhiX libraries at 20 pM, prepared from 2 nM, are stable for up to 3 weeks at − 20 °C. Thus, we strongly recommend that sub-nanomolar libraries less than 25 pM be prepared freshly and sequenced promptly. It should be noted that the aforementioned concentration range was estimated using PhiX DNA. Other libraries may exhibit different applicable ranges due to many factors affecting the cluster density, e.g., insert length, GC content, nucleotide diversity of the first 12 cycles for phasing/prephasing/color matrix corrections, etc. Our second to fifth comparative studies also demonstrated that the sub-nanomolar libraries in a variety of NGS projects yielded robust results comparable to those obtained using the standard Illumina protocol.

It should be noted that although standard Illumina and sub-nanomolar protocols start with different initial library concentration (2 nM vs. 100 pM, respectively), the amount and volume finally loaded onto the sequencer is identical (e.g. 120 μl of 8.3 pM solution, containing 1 fmol of the PhiX library). The main difference is the concentrations of dilution buffers used to produce the 8.3 pM library in the two protocols. By using higher-capacity buffer (200 mM Tris pH 7.0) in the sub-nanomolar protocol, minimal volume (20 μl) of the dilution buffer is introduced, as compared to ~ 1 ml lower-capacity buffer in the Illumina protocol. Since identical volume and amount of the library is loaded onto the sequencer, the potential of introducing method-induced artifacts using our protocol in sequence read quality, mapping results, aberrant splicing events, etc., is probably minimal, if any (Additional file 1: Table S1b, c, d, e). Although we did not have a chance to conduct long non-coding RNA sequencing, there appears no compelling reason to doubt that it could also work for such a workflow as well. In contrast, we found the sub-nanomolar protocol actually performs better than the Illumina protocol in DNA-sequencing of high GC content (68.1%) gDNA samples. Data in Additional file 1: Table S1b show an overall slightly higher # of reads, coverage depth, and # of single nucleotide variants with the sub-nanomolar protocol vs. the Illumina protocol. Whether or not the result has to do with enhanced denaturation using 50 mM NaOH relative to the smaller amount of starting library (1 fmol) in the sub-nanomolar protocol (vs. 50 mM NaOH to 20 fmol library in Illumina protocol) remains to be determined.

An interesting non-linear relationship between the amounts of library loaded and the density of clusters (or no. of reads) was observed. For example, in Fig. 1d, loading one quarter (25%) of a library (2.1 vs. 8.3 pM) led to ~ 55% decrease in cluster density (or no. of reads). This phenomenon, which may account in part for the favorable performance of the sub-nanomolar protocol, might be explainable from the bridge formations during cluster generation under Illumina’s SBS (sequencing by synthesis) sequencing strategy. We envision that the flow cell is covered with a lawn of well-patterned oligonucleotide anchors, onto which single-stranded (denatured) DNA anneals and serves as templates for nucleotide incorporation. Following completion of base extension and removal of templates, newly synthesized strands bend over to those nearby anchoring oligonucleotides and repeat the processes of cluster generation. At lower library concentrations, more nearby oligonucleotides will be available for annealing; forming bridges for the single stranded DNAs would be relatively easy. With increasing concentrations, while the number of bridges formed would increase, the formation of clusters becomes less and less efficient, likely resulting from the competition for non-occupied oligonucleotide anchors at the surrounding sites.

The final concentration of NaOH in Illumina’s suggested protocol is ≤1 mM, equal approximately to pH 11, if not buffered. Illumina’s protocol calls for the addition of 980–990 μl HT1 (proprietary composition), which effectively neutralizes the base and brings the solution pH down to 7–9.5. This step is meant to facilitate single-stranded cDNA hybridization to oligonucleotide anchors on the flow cell. In our sub-nanomolar protocol, the addition of 20 μl of 200 mM Tris (pH 7.0) is to compensate for the lower volume of HT1 added (120 μl in Fig. 1b vs. 980–990 μl HT1 in Fig. 1a). Thus the final pH values (7 to 9.5) of libraries using the sub-nanomolar protocol are in line with Illumina’s protocol. An initial concern over additional salt introduced using 20 μl of 200 mM Tris was later dismissed, as the salt concentration of HT1, after freeze dry, was found to be in the range of 60 mg/ml, making the amount of salt from the 200 mM Tris buffer insignificant.

The steps shown in Fig. 1b generate 120 μl ready-to-load library, an adequate volume for a TruSeq V3 high-output flow cell on the HiSeq 2500. The steps could be scaled up or down to accommodate different loading volumes, e.g., 135 μl (HiSeq rapid mode using cBot), 420 μl (HiSeq rapid mode using on-board clustering), 75 μl (HiSeq V4 flowcell), or 600 μl (MiSeq).

## Conclusions

We studied and validated a robust sub-nanomolar (100 pM) protocol, using PhiX control, genomic DNA, messenger RNA, microRNA, other small noncoding RNA, and 16S rRNA gene amplicons. We further extended the utility of our protocol to library concentrations as low as 25 pM, which generates only slightly less than half of reads achieved with 100 pM. As noted previously, methods allowing application of NGS approaches to samples of limited amounts are needed urgently. Our findings offer investigators the ability to use sub-nanomolar libraries to achieve robust and reliable results comparable to those obtained with the ≥2 nM libraries recommended by the current Illumina MiSeq and HiSeq protocols.

## Additional files

Additional file 1: **Table S1.** A summary of run metrics of the Illumina protocol and the sub-nanomolar protocol. (DOCX 24 kb)

Additional file 2: **Table S2.** Data of variant calling for the three *Bordetella bronchiseptica* RB50 strains and one *Bordetella pertussis* Tohama I strain. (XLSX 122 kb)

Additional file 3: **Table S3.** Highly comparable RPKMs resulted from RNA-Seq using standard Illumina protocol and the sub-nanomolar protocol. (XLSX 1126 kb)

Additional file 4: **Table S4.** Highly comparable RPKMs resulted from MicroRNA-Seq using standard Illumina protocol and the sub-nanomolar protocol. (XLSX 95 kb)

Additional file 5: **Table S5.** Highly comparable RPKMs resulted from other small noncoding RNA-Seq using standard Illumina protocol and the sub-nanomolar protocol. (XLSX 2013 kb)

Additional file 6: **Table S6.** Original pdf outputs from mirDeep2. Data used in Table 1 on IsomiRs (5′ modifications, 3′ modification, and nucleotide substitution) of the miR-34 family activated by p53. (DOCX 68 kb)

Additional file 7: **Table S7.** Highly similar taxonomic hierarchy and relative abundance of classification (sheet 1) and strong correlation of number of hits (sheet 2) resulted form 16S rRNA-Seq using standard Illumina protocol and the sub-nanomolar protocol. (XLSX 40 kb)

## Abbreviations

cBot: Illumina automating cluster generation apparatus
DNA: Deoxyribonucleic acid
HiSeq: Illumina high-throughput sequencer
MiSeq: Illumina high-throughput sequencer
NGS: Next generation sequencing
PCR: Polymerase chain reaction
Phix: an icosahedral, nontailed bacteriophage with a single-stranded DNA
RIN: RNA integrity number
RNA: Ribonucleic acid
