# Supplementary material for: Robust Sub-nanomolar Library Preparation for High Throughput Next Generation Sequencing
Source: BMC Genomics. 2018 May 4;19:326. doi: 10.1186/s12864-018-4677-y (PMC5935984; doi:10.1186/s12864-018-4677-y)
Supplement: Supplementary file 1 — Table S1. A summary of run metrics of the Illumina protocol and the sub-nanomolar protocol. (DOCX 24 kb) [file 12864_2018_4677_MOESM1_ESM.docx]

**Table S1. A summary of run metrics of the Illumina protocol and the sub-nanomolar protocol.**

1. DNA-sequencing using PhiX.

|  | Cluster density (K/mm2) | Cluster PF (%) | Reads PF (millions) | Aligned (%) | Error Rate (%) | % of bases ≥ Q30 |
| --- | --- | --- | --- | --- | --- | --- |
| Illumina protocol, 2 nM → 8.3 pM | 557 | 95.87 | 147.63 | 99.3 | 0.21 | 97.8 |
| Sub-nanomolar, 100 pM → 8.3 pM | 542 | 95.75 | 143.35 | 99.2 | 0.22 | 97.8 |
| Sub-nanomolar, 80 pM → 6.7 pM | 484 | 96.26 | 128.80 | 99.3 | 0.23 | 98.0 |
| Sub-nanomolar, 50 pM → 4.2 pM | 313 | 96.63 | 83.64 | 98.3 | 0.26 | 98.0 |
| Sub-nanomolar, 40 pM → 3.3 pM | 268 | 97.15 | 71.93 | 99.3 | 0.19 | 98.8 |
| Sub-nanomolar, 25 pM → 2.1 pM | 248 | 97.35 | 66.71 | 99.3 | 0.20 | 98.8 |

1. Genomic DNA-sequencing using three *Bordetella bronchiseptica* RB 50 strain (1739, 1919, 1920) and one *Bordetella pertussis* Tohama I strain (420) with additional mapping and variant calling data.

| All at 10 pM loading. | # of reads (million) | Coverage depth (x) | Aligned (%) | % of bases ≥ Q30† | # of SNVs* | # of Insertions | # of deletions |
| --- | --- | --- | --- | --- | --- | --- | --- |
| Illumina protocol, strain 1739 | 1,522,362 | 42 | 98.9% | 89.2% | 20 | 0 | 3 |
| Illumina protocol, strain 1919 | 1,550,506 | 43 | 98.9% | 90.2% | 12 | 1 | 3 |
| Illumina protocol, strain 1920 | 1,610,206 | 44 | 97.8% | 89.5% | 23 | 0 | 2 |
| Illumina protocol, strain 420 | 2,433,640 | 87 | 97.6% | 89.8% | 401 | 22 | 19 |
| **Illumina protocol, overall** | **7,116,714 (sum)** | **54 (avg)** | **98.3% (avg)** | **89.7% (avg)** | **456 (sum)** | **23 (sum)** | **27 (sum)** |
| Sub-nanomolar, strain 1739 | 1,220,194 | 34 | 98.8% | 87.8% | 29 | 0 | 3 |
| Sub-nanomolar, strain 1919 | 2,381,772 | 66 | 98.6% | 89.8% | 25 | 1 | 2 |
| Sub-nanomolar, strain 1920 | 2,158,566 | 59 | 97.7% | 88.8% | 28 | 0 | 3 |
| Illumina protocol, strain 420 | 2,208,998 | 79 | 97.4% | 88.8% | 421 | 21 | 20 |
| **Sub-nanomolar, overall** | **7,969,530 (sum)** | **59 (avg)** | **98.1% (avg)** | **88.8% (avg)** | **503 (sum)** | **22 (sum)** | **28 (sum)** |

*SNVs: single nucleotide variants. †from bcl2fastq’s laneBarcode.html.

1. mRNA-sequencing using HCT 116 and HCoEpiC cells.

| All at 8.3 pM loading. | # of reads (millions) | Aligned (%) | % of bases ≥ Q30† |
| --- | --- | --- | --- |
| Illumina protocol, HCT 116 | 27,909,366 | 84.2% | 80.1% |
| Sub-nanomolar, HCT 116 | 28,940,758 | 82.9% | 78.9% |
| Illumina protocol, HCoEpiC | 30,468,484 | 83.9% | 78.7% |
| Sub-nanomolar, HCoEpiC | 31,333,756 | 82.6% | 77.5% |

†from bcl2fastq’s laneBarcode.html.

1. MicroRNA-sequencing using HCoEpiC cells.

| All at 8.3 pM loading. | # of reads (millions) | Aligned (%) | % of bases ≥ Q30† |
| --- | --- | --- | --- |
| Illumina protocol, HCoEpiC | 6,998,358 | 83.2% | 97.4% |
| Sub-nanomolar, HCoEpiC | 4,237,130 | 86.1% | 97.4% |

†from bcl2fastq’s laneBarcode.html.

1. 16S metagenomic sequencing using staggered microbial mock community B from Bei Resources.

| All at 4 pM loading. | # of reads (millions) | Aligned (%) | % of bases ≥ Q30† |
| --- | --- | --- | --- |
| Illumina protocol | 2,249,926 | n/a | 62.9% |
| Sub-nanomolar | 2,148,506 | n/a | 61.2% |

†from bcl2fastq’s laneBarcode.html.
