# Supplementary material for: Robust Sub-nanomolar Library Preparation for High Throughput Next Generation Sequencing
Source: BMC Genomics. 2018 May 4;19:326. doi: 10.1186/s12864-018-4677-y (PMC5935984; doi:10.1186/s12864-018-4677-y)
Supplement: Supplementary file 6 — Table S6. Original pdf outputs from mirDeep2. Data used in Table 1 on IsomiRs (5′ modifications, 3′ modification, and nucleotide substitution) of the miR-34 family activated by p53. (DOCX 68 kb) [file 12864_2018_4677_MOESM6_ESM.docx]

Table S6. Original pdf outputs from mirDeep2. Data used in Table 1 on IsomiRs (5' modifications, 3' modification, and nucleotide substitution) of the miR-34 family activated by p53.

Double click to open:

| hsa-miR-34a |  |
| --- | --- |
| hsa-miR-34b |  |
| hsa-miR-34c |  |
